# Telephone calls to patients after discharge from the hospital: an important part of transitions of care

**DOI:** 10.3402/meo.v20.26701

**Published:** 2015-04-29

**Authors:** Janet D. Record, Ashwini Niranjan-Azadi, Colleen Christmas, Laura A. Hanyok, Cynthia S. Rand, David B. Hellmann, Roy C. Ziegelstein

**Affiliations:** 1 Department of Medicine, Johns Hopkins Bayview Medical Center, Johns Hopkins University School of Medicine, Baltimore, MD, USA; 2 Department of Medicine, Johns Hopkins Hospital, Johns Hopkins University School of Medicine, Baltimore, MD, USA

**Keywords:** patient-centered care, graduate medical education, post-discharge telephone call, care transitions

## Abstract

**Background:**

Teaching interns patient-centered communication skills, including making structured telephone calls to patients following discharge, may improve transitions of care.

**Objective:**

To explore associations between a patient-centered care (PCC) curriculum and patients’ perspectives of the quality of transitional care.

**Methods:**

We implemented a novel PCC curriculum on one of four inpatient general medicine resident teaching teams in which interns make post-discharge telephone calls to patients, contact outpatient providers, perform medication adherence reviews, and engage in patient-centered discharge planning. Between July and November of 2011, we conducted telephone surveys of patients from all four teaching teams within 30 days of discharge. In addition to asking if patients received a call from their hospital physician (intern), we administered the 3-Item Care Transitions Measure (CTM-3), which assesses patients’ perceptions of preparedness for the transition from hospital to home (possible score range 0–100).

**Results:**

The CTM-3 scores (mean±SD) of PCC team patients and standard team patients were not significantly different (82.4±17.3 vs. 79.6±17.6, *p*=0.53). However, regardless of team assignment, patients who reported receiving a post-discharge telephone call had significantly higher CTM-3 scores than those who did not (84.7±16.0 vs. 78.2±17.4, *p*=0.03). Interns exposed to the PCC curriculum called their patients after discharge more often than interns never exposed (OR=2.78, 95% CI [1.25, 6.18], *p*=0.013).

**Conclusions:**

The post-discharge telephone call, one element of PCC, was associated with higher CTM-3 scores – which, in turn, have been shown to lessen patients’ risk of emergency department visits within 30 days of discharge.

Although the Institute of Medicine has highlighted the importance of patient-centered care (PCC) to health care quality (1), explicit training in patient-centered communication skills remains absent from most residency training programs (2). This is especially unfortunate, because patients, during the early post-discharge period, are at risk for developing new or worsening symptoms and may exhibit incomplete understanding of instructions for self-management (3, 4). Providing PCC during a hospitalization, coupled with a telephone call in the early post-discharge period, may mitigate these risks.

We developed and implemented a PCC curriculum for internal medicine residents that emphasizes patient-centered communication skills, including post-discharge telephone contact, to achieve safer transitions of care (5). Previously, this same curriculum was shown to be associated with reduced 30-day heart failure readmissions (6), improved patient satisfaction (7), and improvements in residents’ self-assessed ability to communicate with patients about medications and discharge transitions (7). This study, then, extends this line of inquiry by examining whether patients discharged from the inpatient medicine team utilizing the PCC curriculum would report a higher quality transition of care out of the hospital. We also examined the relationship between post-discharge telephone calls, an important component of the PCC curriculum, and patients’ perceptions of care transitions.

## Methods

The study setting was the inpatient general medicine teaching service at Johns Hopkins Bayview Medical Center – a 335-bed, urban academic medical center. In this observational cohort study, one of four inpatient general medicine resident teaching teams (one attending, one resident, two interns, and two medical students) utilized the PCC curriculum. Because patients were assigned to teaching teams based on a rotating call schedule, specific patient characteristics (e.g., illness acuity) played no role in team assignment.

On the intervention team utilizing the PCC curriculum, interns learned and had primary responsibility for the practice of effective post-discharge telephone calls to patients, contact with outpatient providers, medication adherence reviews, and patient-centered discharge planning (5). The post-discharge calls followed the SCOTCH structure: 1) *S*et up (ask whether the patient is ready to talk); 2) *C*heck the patient's understanding of the hospitalization; 3) ask about *O*pportunities for the medical team to improve; 4) ask how the *T*ransition home went; 5) *C*heck the patient's understanding of recommendations for ongoing care; and 6) offer to *H*elp as needed (5).

While rotating on standard teams, interns were not explicitly asked to perform any of the specific PCC-related activities, although some had been previously exposed to the PCC curriculum. Between July and November of 2011, a study team member contacted patients discharged home within 30 days to screen for eligibility. Patients were excluded if they: 1) did not speak English; 2) were unable to participate due to hearing difficulties; 3) were incapable of providing consent; 4) were in hospice, discharged against medical advice, or rehospitalized; 5) had died; or 6) lacked telephone access. Callers were blinded to patients’ team assignment, were not involved in their care, and closely followed a standardized script.

The survey began with the 3-Item Care Transitions Measure (CTM-3), which assesses the degree to which patients agree that the hospital staff considered their preferences, clarified responsibilities in self-management, and explained the purpose for taking each medication. CTM-3 scores are reported on a 100-point scale – with higher scores representing higher ratings of transitional care (8). For each of the three CTM questions, answer choices included strongly disagree (1 point), disagree (2 points), agree (3 points), and strongly agree (4 points). As per standard instructions for use of the CTM-3 questionnaire, the steps in generating a patient's total CTM-3 score include calculating a mean item score, and then transforming this mean item score to a 100-point scale, using the formula [(mean item score – 1)÷3]×100. If a patient does not answer a CTM-3 question, the available answers are used to calculate a mean item score, based on the number of items answered. We chose the CTM-3 measure because it: 1) produces (in comparable contexts) valid scores of patients’ perceived preparedness for their care transition; 2) predicts need for emergency department (ED) visits within 30 days of discharge (9); 3) is endorsed by the National Quality Forum (10); and 4) is less burdensome than the 15-item version from which it derives (8). We also asked patients, ‘Did you feel that your doctor(s) really knew you as a person?’ (yes/somewhat/no/don't know), and ‘Did your hospital doctor call you after you left the hospital?’ (yes/no/don't know).

Mean CTM-3 scores were compared using independent *t-*tests, and categorical variables were compared using chi-square statistics. Statistical significance was set at *p*≤0.05. A Johns Hopkins Institutional Review Board reviewed our study and determined it constituted program evaluation and therefore did not require ongoing oversight.

## Results

During the study period, 12 and 26 interns rotated on the PCC and standard teams, respectively – discharging 239 and 1,190 patients. Surveys were completed for a total of 139 (9.7%) patients: 18 (7.5%) discharged from the PCC team and 121 (10.2%) from the 3 standard teams. In order to complete 139 calls, we attempted to contact 289 patients (44 on PCC team, 245 on standard teams); 18 patients declined to participate (2 on PCC team, 16 on standard teams); the remainder of incomplete calls were due to patients being unreachable by telephone or due to hearing or cognitive limitations. Thus, the survey response rate was 48.1% overall, 40.9% on the PCC team, and 49.4% on the standard teams. CTM-3 scores ranged from 11.1 to 100 overall, from 50 to 100 on the PCC team, and from 11.1 to 100 on the standard teams. Mean CTM-3 scores (mean±SD) were 82.4±17.3 for PCC patients, and 79.6±17.6 for those of the standard teams – a non-significant difference (*t*=0.63, *df*=137, *p*=0.53). As expected, the majority of patients (82.4%) cared for on the PCC team reported receiving a post-discharge telephone call; 29.5% on the standard teams also reported receiving a call (*X*^2^=18.09, *df =*1, *p*≤0.001). The proportion of patients receiving follow-up calls from interns ever versus never exposed to the PCC curriculum was significantly larger – 54.5% versus 30.2% (*X*^2^=6.48, *df*=1, *p*=0.01), respectively. Lastly, regardless of team assignment, patients who reported receiving post-discharge calls had significantly higher CTM-3 scores – 84.7±16.0 versus 78.2±17.4 (*t*=2.16, *df*=137, *p*=0.03).

## Discussion

In this study, patients discharged from the PCC and standard teams reported a similar quality of transitional care based on CTM-3 scores. However, regardless of team assignment, patients who reported receiving a post-discharge telephone call had significantly higher CTM-3 scores compared to those who received no such follow-up. Interns exposed to the PCC curriculum called their patients more often after hospital discharge than interns never exposed (OR = 2.78, 95% CI [1.25, 6.18], *p*=0.013).

The absence of a difference in CTM-3 scores between patient care teams may be explained by several factors. First, the sample size was small, limiting the power to detect a difference. Additionally, some members of standard teams had previously been exposed to the PCC curriculum as interns, which may have led to a diffusion of PCC concepts and behaviors. Of note, CTM-3 mean scores were fairly high – ranging in other studies from 64 to 83 (8, 11, 12).

Patients who received a post-discharge call from their hospital physician (intern) reported significantly higher CTM-3 scores. In another study, a similar absolute difference in mean CTM-15 scores distinguished patients who did and did not require subsequent ED visits and hospital readmissions for the same principal diagnosis after hospital discharge (13).

Given that patients are vulnerable soon after leaving the hospital, post-discharge telephone calls may improve clinical outcomes; however, this is not the current standard of care (14). A Cochrane review of hospital-based telephone follow-ups after discharge from hospital to home found clinically equivalent results in the intervention and control groups (15). Some studies in this review showed improvement in outcomes such as knowledge, adherence, satisfaction, ED visits, and readmissions (16–20); other studies found no changes in these outcomes (21–23). However, most prior studies have evaluated impact of calls made by nurses or pharmacists who were not directly involved with care of the patients during hospitalization (15–23). In our intervention, a physician member of the patient's hospital care team made the post-discharge telephone call.

Other studies examining the impact of scripted, post-discharge calls from nurses at patients’ primary care clinics (24, 25) found that new symptoms and medication concerns were among the most frequent problems identified and addressed (24). Balaban and associates found that patients receiving a call were more likely to follow up in the primary care office within 21 days of discharge and to receive the follow-up tests and evaluation recommended by the inpatient team (25). However, neither study found reductions in readmission rates or in ED visits. Moreover, generalizability of these studies was limited, given their focus on patients with established primary care prior to the hospitalization.

The structure of telephone calls explicitly taught in our PCC curriculum emphasized use of open-ended questions and checking patient understanding. This patient-centered approach may help explain the association between receiving a call and perceiving better preparedness for discharge home. Although the optimal content and structure of a post-discharge phone call has not been established (26), we believe the exploration of patients’ perspectives, concerns, and understanding are strengths of our approach.

The odds of an intern calling a patient after discharge were nearly three times greater for interns ever exposed to the PCC curriculum, regardless of team assignment. The prevalence of this behavior on standard teams may suggest that interns perceived value in conducting these calls.

Limitations of this study include a small sample size, a single-institution setting, and a non-randomized design. Given the myriad of potential confounds, we cannot establish a causal effect of the telephone call; for example, interns who made post-discharge telephone calls may have also engaged in other behaviors that enhanced patients’ transitions home. In our survey, we asked patients to recall whether they received a call from their hospital physician after discharge; we were unable to verify whether some of these remembered phone calls may have been from other health system employees gathering data about patients’ recent hospital care. Patients who lacked access to a working telephone were unable to benefit from a post-discharge telephone call or participate in the study, and we were unable to fully control for potential differences in illness acuity or frequency of recent hospital admission between intervention and comparison group patients. Finally, it is possible that the population sampled is not entirely representative of all patients who would have been eligible for the study.

## Conclusion

Although we cannot determine causality, a physician-initiated, post-discharge telephone call, one element of PCC, was associated with higher scores on the CTM-3, which in turn predict a lower risk of requiring ED visits within 30 days of discharge (9). Whether routine, structured post-discharge telephone calls by physicians would ultimately result in enhanced patient care warrants further study.

## References

[1] Defining the contents of the data set: the national health care quality framework. Envisioning the national healthcare quality report, 2001.

[2] Levinson W, Lesser CS, Epstein RM (2010). Developing physician communication skills for patient-centered care. Health Aff (Millwood).

[3] Epstein K, Juarez E, Loya K, Gorman MJ, Singer A (2007). Frequency of new or worsening symptoms in the post hospitalization period. J Hosp Med.

[4] Calkins DR, Davis RB, Reiley P, Phillips RS, Pineo KL, Delbanco TL (1997). Patient-physician communication at hospital discharge and patients’ understanding of the post discharge treatment plan. Arch Intern Med.

[5] Hanyok L, Brandt L, Christmas C, Hellmann D, Rand C, Ratanawongsa N (2012). The Johns Hopkins Aliki initiative: a patient-centered curriculum for internal medicine residents. MedEdPORTAL.

[6] Record JD, Rand C, Christmas C, Hanyok L, Federowicz M, Bilderback A (2011). Reducing heart failure readmissions by teaching patient-centered care to internal medicine residents. Arch Intern Med.

[7] Ratanawongsa N, Federowicz MA, Christmas C, Hanyok LA, Record JD, Hellmann DB (2012). Effects of a focused patient-centered care curriculum on the experiences of internal medicine residents and their patients. J Gen Intern Med.

[8] Parry C, Mahoney E, Chalmers SA, Coleman EA (2008). Assessing the quality of transitional care: further applications of the care transitions measure. Med Care.

[9] Coleman EA, Parry C, Chalmers SA, Chugh A, Mahoney E (2007). The central role of performance measurement in improving the quality of transitional care. Home Health Care Serv Q.

[10] 3-Item Care Transition Measure (CTM-3) Measures. National Quality Forum.

[11] Arbaje AI, Maron DD, Yu Q, Wendel VI, Tanner E, Boult C (2010). The geriatric floating interdisciplinary transition team. J Am Geriatr Soc.

[12] Sides EG, Zimmer LO, Wilson L, Pan W, Olson DM, Peterson ED (2012). Medication coaching program for patients with minor stroke or TIA: a pilot study. BMC Public Health.

[13] Coleman EA, Mahoney E, Parry C (2005). Assessing the quality of preparation for posthospital care from the patient's perspective: the care transitions measure. Med Care.

[14] Centers for Medicare & Medicaid Services Quality measures and performance standards. http://www.webcitation.org/6UG8mwvFn.

[15] Mistiaen P, Poot E (2006). Telephone follow-up, initiated by a hospital-based health professional, for postdischarge problems in patients discharged from hospital to home. Cochrane Database Syst Rev.

[16] Beckie T (1989). A supportive-educative telephone program: impact on knowledge and anxiety after coronary artery bypass graft surgery. Heart Lung.

[17] Ritchie PD, Jenkins M, Cameron PA (2000). A telephone call reminder to improve outpatient attendance in patients referred from the emergency department: a randomised controlled trial. Aust N Z J Med.

[18] Braun E, Baidusi A, Alroy G, Azzam ZS (2009). Telephone follow-up improves patients satisfaction following hospital discharge. Eur J Intern Med.

[19] Dudas V, Bookwalter T, Kerr KM, Pantilat SZ (2001). The impact of follow-up telephone calls to patients after hospitalization. Am J Med.

[20] Kind AJ, Jensen L, Barczi S, Bridges A, Kordahl R, Smith MA (2012). Low-cost transitional care with nurse managers making mostly phone contact with patients cut rehospitalization at a VA hospital. Health Aff (Millwood).

[21] Barnason S, Zimmerman L (1995). A comparison of patient teaching outcomes among postoperative coronary artery bypass graft (CABG) patients. Prog Cardiovasc Nurs.

[22] Nelson EW, Van Cleve S, Swartz MK, Kessen W, McCarthy PL (1991). Improving the use of early follow-up care after emergency department visits. A randomized trial. Am J Dis Child.

[23] Tranmer JE, Parry MJ (2004). Enhancing postoperative recovery of cardiac surgery patients: a randomized clinical trial of an advanced practice nursing intervention. West J Nurs Res.

[24] Tang N, Fujimoto J, Karliner L (2014). Evaluation of a primary care-based post-discharge phone call program: keeping the primary care practice at the center of post-hospitalization care transition. J Gen Intern Med.

[25] Balaban RB, Weissman JS, Samuel PA, Woolhandler S (2008). Redefining and redesigning hospital discharge to enhance patient care: a randomized controlled study. J Gen Intern Med.

[26] Crocker JB, Crocker JT, Greenwald JL (2012). Telephone follow-up as a primary care intervention for postdischarge outcomes improvement: a systematic review. Am J Med.

